# Assessing Sodium Intake in Middle-Aged and Older Adults with Elevated Blood Pressure: Validation of Spot Urine Excretion and Dietary Survey-Derived Estimates

**DOI:** 10.3390/nu16101461

**Published:** 2024-05-13

**Authors:** Yee Chang Soh, Andrea Fairley, Mawada Alawad, Siew Siew Lee, Tin Tin Su, Blossom Christa Maree Stephan, Daniel Reidpath, Louise Robinson, Shajahan Yasin, Mario Siervo, Devi Mohan

**Affiliations:** 1 Global Public Health, Jeffrey Cheah School of Medicine and Health Sciences, Monash University Malaysia, Bandar Sunway 47500, Malaysia; yee.soh@monash.edu (Y.C.S.); mawada.alawad@monash.edu (M.A.); tintin.su@monash.edu (T.T.S.); 2 South East Asia Community Observatory (SEACO), Jeffrey Cheah School of Medicine and Health Sciences, Monash University Malaysia, Bandar Sunway 45700, Malaysia; 3 School of Biomedical, Nutritional and Sports Sciences, Newcastle University, Newcastle upon Tyne NE2 4DR, UK; andrea.fairley@newcastle.ac.uk; 4 School of Biosciences, Faculty of Science and Engineering, University of Nottingham Malaysia, Semenyih 43500, Malaysia; lee.siew@nottingham.edu.my; 5 Institute of Mental Health, School of Medicine, University of Nottingham, Nottingham NG7 2TU, UK; blossom.stephan@nottingham.ac.uk; 6 Dementia Centre of Excellence, Curtin enAble Institute, Faculty of Health Sciences, Curtin University, Perth, WA 6845, Australia; 7 Institute for Global Health and Development, Queen Margaret University, Musselburgh EH21 6UU, UK; dreidpath@qmu.ac.uk; 8 School of Social Sciences, Monash University, Melbourne, VIC 3800, Australia; 9 Population Health Sciences Institute, Newcastle University, Newcastle upon Tyne NE4 5PL, UK; a.l.robinson@newcastle.ac.uk; 10 Jeffrey Cheah School of Medicine and Health Sciences, Monash University Malaysia, Bandar Sunway 47500, Malaysia; shah.yasin@monash.edu; 11 School of Population Health, Faculty of Health Sciences, Curtin University, Perth, WA 6845, Australia; mario.siervo@curtin.edu.au; 12 School of Public Health, The University of Queensland, Herston, QLD 4006, Australia

**Keywords:** sodium intake assessment, 24-h urine, spot urine, 24-h dietary recall, food frequency questionnaire, elevated blood pressure, hypertension

## Abstract

This cross-sectional study evaluated the validity of three alternative methods compared to the gold standard 24-h urine collection for estimating dietary sodium intake, a modifiable risk factor for hypertension, among middle-aged and older adults with elevated blood pressure. These included spot urine collection (using Kawasaki, Tanaka, and INTERSALT equations), 24-h dietary recall, and food frequency questionnaire responses, compared to 24-h urine collection in a subset of 65 participants (aged 50–75 years, 58.5% women, 61.6% hypertensive) from the DePEC-Nutrition trial. The validity of the methods was assessed using bias, the Spearman correlation coefficient (SCC), the intraclass correlation coefficient (ICC), and Bland–Altman analysis. Among the alternative methods, spot urine collection using the Kawasaki equation showed the strongest correlation (SCC 0.238; ICC 0.119, 95% CI −0.079 to 0.323), but it exhibited a significant bias (1414 mg/day, *p*-value < 0.001) relative to 24-h urine collection. Conversely, dietary surveys had a smaller bias but wider limits of agreement. These findings underscore the complexities of accurately estimating dietary sodium intake using spot urine collection or dietary surveys in this specific population, suggesting that a combination or the refinement of existing methodologies might improve accuracy. Further research with larger samples is necessary to develop more reliable methods for assessing sodium intake in this high-risk group.

## 1. Introduction

Elevated blood pressure (BP) is categorized into prehypertension with systolic blood pressure (SBP) between 120 and <140 mmHg or diastolic blood pressure (DBP) between 80 and <90 mmHg and hypertension (HPT) with SBP of ≥140 or DBP of ≥90 mmHg or the use of antihypertensive medication. Individuals with prehypertension are at increased risk of developing HPT [1]. Globally, HPT is a significant public health concern, as it is closely associated with an increased risk of cardiovascular diseases, including heart attacks and strokes, as well as other health complications [2]. One key modifiable risk factor in the development and management of hypertension is dietary sodium intake [3]. Given its modifiability, sodium intake becomes a critical target for preventive measures aimed at reducing the incidence and severity of HPT. However, accurately assessing an individual’s sodium intake poses substantial challenges, particularly when dealing with adults who already have elevated BP. This challenge arises from various factors, with one notable consideration being the impact of antihypertensive drugs on sodium excretion [4]. Antihypertensive medications, which are commonly prescribed to lower BP level patients, can influence the way the body handles sodium. These drugs may enhance the elimination of sodium through urine or affect sodium retention in the body, making it difficult to precisely gauge an individual’s sodium intake solely based on conventional dietary assessments. This becomes particularly important for middle-aged and older adults who are often on medication for BP management [5]. Accurate measurement of sodium intake in this demographic is crucial for the design of effective dietary interventions and the evaluation of sodium reduction strategies.

Approximately 93% of dietary sodium is excreted in urine, making 24-h urine collection the gold standard for estimating sodium intake at both the population and individual levels [6]. However, this method has limitations, including being time-consuming, costly, and challenging to implement in community and low- or middle-income country (LMIC) settings, often leading to recruitment and dropout issues [6,7,8]. Alternative methods, such as spot urine collection, 24-h dietary recall (24DR), and the food frequency questionnaire (FFQ), have been developed and used to estimate sodium intake.

Spot urine collection, common in epidemiological studies, offers a practical and cost-effective way to estimate sodium intake. However, it assumes proportional sodium excretion in spot urine samples compared to 24-h excretion. Equations like Kawasaki, Tanaka, and INTERSALT aim to improve accuracy but tend to overestimate at lower excretion levels and underestimate at higher levels [9,10,11,12].

Estimating sodium intake through a 24DR involves a detailed interview where individuals report all foods and beverages consumed in the past 24 h. This method requires precise descriptions of portion sizes and preparation methods. Subsequently, food composition databases or software are used to look up the sodium content of each reported item. This includes accounting for sodium-containing seasonings, condiments, and added salt during cooking or at the table (discretionary sources). By summing up the sodium content of all items, an estimate of the total sodium intake for the 24-h period is derived. However, it relies on memory and reporting accuracy, making it susceptible to bias and portion estimation errors, especially for hidden sodium sources such as canned vegetables, bread products, cereal and grains, meat products, and dairy products [13].

The FFQ is a self-administered questionnaire that captures an individual’s typical dietary habits over an extended period, often a month or year. Respondents indicate how frequently they consume various foods, including those containing sodium, and specify their usual portion sizes. Estimating sodium intake from the FFQ involves assigning an estimated sodium content to each food item based on standard portion sizes and reported sodium levels. Multiplying the consumption frequency by the estimated sodium content for each food item allows for calculating the total sodium intake over the specified period. Although the FFQ provides insights into long-term dietary patterns and is less burdensome than 24-h urine collection, it also has limitations, including potential inaccuracies related to self-reporting and the need for reliable sodium content data. This makes it less effective for capturing daily variations [13,14].

While the gold standard for assessing sodium intake involves the high participant burden and resource-intensive 24-h urine collection method, the performance of alternative methods remains underexplored in middle-aged and older adults with elevated BP. Dietary surveys exhibit variations in food databases and data collection across studies and populations. Moreover, high measurement validity is crucial when linking estimated sodium intake to clinical outcomes.

In Malaysia, the prevalence of HPT among adults is notably high, with a study indicating a rate of 49.4%, which contrasts with the national average of 35.3% [15]. This discrepancy highlights the rising concern of hypertension alongside modernization and lifestyle changes, including high intake of salty and fatty foods. Efforts to manage HPT in Malaysia involve salt reduction strategies to curb the average daily sodium intake, addressing a significant risk factor for elevated BP. The standard 24-h urine collection method, while effective, poses significant challenges in Malaysia, a LMIC. This situation highlights the necessity for developing alternative, practical methods for sodium intake assessment that are suitable for the Malaysian context and other similar LMIC settings.

This study aimed to evaluate the relative validity of three alternative methods, including spot urine collection (using the Kawasaki, Tanaka, and INTERSALT equations), 24DR, and FFQ, against the gold standard in a cohort of middle-aged and older Malaysian adults with elevated BP. It was hypothesized that these alternative methods can provide a valid estimates of sodium intake in this specific population, potentially offering more feasible options for dietary sodium assessment in similar contexts globally.

## 2. Materials and Methods

### 2.1. Study Design

This cross-sectional validation study was conducted within the larger DePEC (Dementia Prevention and Enhanced Care)-Nutrition trial. DePEC-Nutrition is a 2 × 2 factorial feasibility trial aimed at implementing a dietary intervention to reduce salt intake and increase high-nitrate vegetable consumption over 24 weeks. This intervention targeted community-dwelling middle-aged and older adults with prehypertension and stage I hypertension, with the overarching goal of preventing cognitive decline in this population. Participants in the validation study were recruited from the DePEC-Nutrition trial and required to meet the following inclusion criteria: (1) being aged 50–75 years old; (2) residing within a 5 km radius of the study site; (3) SBP of 120–159 mmHg, diastolic blood pressure of 80–99 mmHg, or being diagnosed as hypertensive; and (4) a body mass index (BMI) > 18.5 kg/m^2^. This study received ethical approval from both the Monash University Human Research Committee (Project ID: 17864; 15 January 2019) and the Malaysian Medical Research Ethics Committee (NMRR-19-617-45916; 30 September 2019). The protocol was registered with the clinical trial registry (ISRCTN47562685). Detailed information on the study design and participant recruitment for the DePEC-Nutrition trial can be found in previous publications [16,17].

### 2.2. Study Procedure

Participants in the validation study were drawn from those who attended the baseline visit of the DePEC-Nutrition trial. All 74 participants who presented were considered eligible and provided informed consent. Comprehensive baseline data were collected, including sociodemographics; comorbidities, resting blood pressure, anthropometry, cognitive status were assessed via the Malay version of the Mini-Mental State Examination (M-MMSE-7); and physical activity levels were assessed via the International Physical Activity Questionnaire (IPAQ). Detailed descriptions of these assessment methods have been published [16].

#### 2.2.1. Assessment of Dietary Sodium Intake

Participants’ dietary sodium intakes were assessed using four different methods: 24-h urine collection, spot urine collection, a 1-day 24DR, and a FFQ. The 24-h urine collection was designated as the reference method. Eligible participants were given detailed instructions on how to collect their 24-h urine samples using the provided kits, and they were asked to return their samples to the clinic the following morning after the completion of the collection period. Concurrently with this collection, spot urine samples were obtained. The 24DR was conducted by a trained interviewer, who guided participants through recalling all food and beverage intake from the preceding day, coinciding with their urine collection. The FFQ, aimed at capturing dietary habits over the past month, was self-administered by the participants at home.

**Figure 1:**
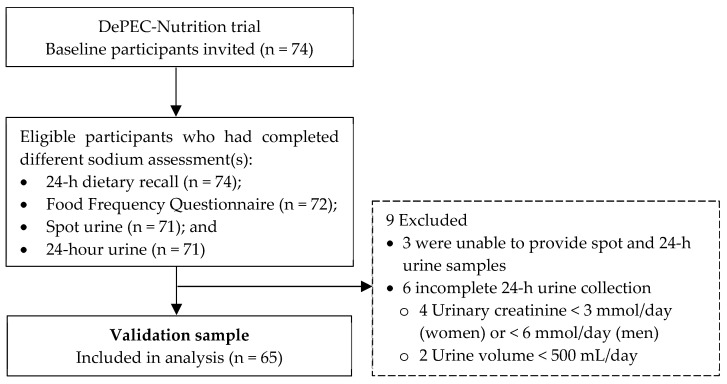
Flowchart on the selection of validation sample.

Of the 74 eligible participants, all successfully completed the 1-day 24DR. A total of 72 participants (97.3% response rate) completed the FFQ, and 71 (95.9% response rate) retuned their 24-h urine samples to the clinic. Another 71 participants (95.9% response rate) provided spot urine samples. Detailed information regarding the procedures for collecting 24-h and spot urine samples can be found elsewhere [13]. The completeness of the 24-h urine samples was determined based on the duration of urine collection, total urine volume, and 24-h urinary creatinine excretion. Nine participants were excluded due to being unable to provide the spot or urine sample or incomplete 24-h urine samples, which were characterized by urinary creatinine excretion below 3 mmol/day for women and 6 mmol/day for men, or a total urine volume of less than 500 mL/day. Consequently, 65 participants with complete data across all four assessment methods were included in the validation study (Figure 1).

#### 2.2.2. Analysis of Dietary Sodium Intake

In the laboratory, sodium concentrations in the 24-h urine and spot urine samples were determined using the indirect ion-selective electrode method with a 1:31 dilution ratio, executed on an Architect c8000 analyzer (Abbott Laboratories, Abbott Park, IL, USA). To verify the completeness of the 24-h urine collection, 24-h urinary creatinine excretion was measured by employing the enzymatic assay method on the same instrument. Total 24-h urinary sodium excretion was used as a surrogate marker of daily sodium intake. Given that approximately 93% of dietary sodium is excreted in the urine, with the remainder eliminated via skin and feces [4], we corrected for this by multiplying the urine volume (L/24-h) by the sodium concentration (mmol/L) by the atomic weight of sodium (23 mg/mmol) and applying a correction factor of 100/93 to estimate daily sodium intake in mg/24 h. This value was then converted to salt intake using a factor of 2.542.

To estimate 24-h urinary sodium excretion from spot urine samples, the Tanaka, Kawasaki, and INTERSALT equations were applied. The Tanaka and Kawasaki equations, developed based on Japanese cohorts, infer that the sodium-to-creatinine ratio in spot samples mirrors those of 24-h samples, allowing for predicted creatinine excretion. These equations were selected due to their derivation from an Asian population, mirroring our study’s context. The INTERSALT equation, derived from a diverse population sample, utilizes regression analysis with spot urinary sodium as a predictor.

Dietary sodium intake estimations from the 1-day 24DR and FFQ were performed using Nutritionist Pro software version 7.5 (Axxya Systems LLC., Redmond, WA, USA), primarily referencing the Malaysian Food Composition Database (MyFCD) [18]. In cases where a specific food item was unavailable in MyFCD, alternative databases were employed, including the United States Department of Agriculture (USDA) standard reference database, the Canadian Nutrient File, and the Food and Nutrient Database for Dietary Study. Standard recipes were employed for composite dishes not listed.

For the 24DR, the precise descriptions of portion sizes and preparation methods of the foods and beverages consumed over the past 24 h, as reported by the individuals, were utilized. Using the food composition databases and software, the sodium content of each reported item, including sodium-containing seasonings, condiments, and added salt during cooking or at the table (discretionary sources), was determined. The analysis of participants’ dietary sodium intake estimated through the 1-day 24DR was conducted using Nutritionist Pro software version 7.5 (Axxya Systems LLC., Redmond, Washington, USA). The primary reference database for estimating sodium intake was the Malaysian Food Composition Database (MyFCD). In cases where a specific food item was unavailable in MyFCD, other databases such as the Singapore Energy and Nutrient Composition of Food, the United States Department of Agriculture (USDA) standard reference database, the Canadian Nutrient File, and the Food and Nutrient Database for Dietary Study, were referenced. Composite dishes not found in these databases were incorporated into the software by including standard recipes for the respective foods. By summing up the sodium content of all items, an estimate of the total sodium intake for the 24-h period was derived.

For estimating sodium intake from the FFQ, an estimated sodium content was assigned to each food item based on standard portion sizes and reported sodium levels from the food composition databases. Similarly, the primary reference database for sodium intake estimation was the MyFCD. To calculate daily sodium intake over the one-month period, the daily amount of consumption (frequency × number of servings × standard portion size) was multiplied by the standard sodium value (derived from the reported sodium levels) for each food item.

### 2.3. Development and Administration of 24DR Form

The 24DR form used in this validation study was adapted from the questionnaire employed in the Malaysian National Health and Morbidity Survey (MANS) conducted in 2014 [19]. Several modifications were made: (1) the inclusion of questions to capture information about special occasions on the 24DR day, as this event could affect the habitual dietary intake; (2) the incorporation of questions regarding the quantity of leftover food and second helpings of the main meal, as these details are often overlooked; and (3) the addition of a checklist encompassing various food and beverage categories that participants might have consumed between meals or as snacks.

During the interview, participants were asked to provide comprehensive information, including the type of food consumed, cooking methods employed, estimated portion sizes, and brand information, for both food and beverages consumed during the preceding 24-h period. Neutral prompts and probes were used throughout the interview session to prevent the biased responses of participants. To aid participants in determining portion sizes during the interview, a standard local household utensil set comprising glasses, cups, bowls, plates, and spoons was used. Visual aids in the form of images of household measures were sourced from a local food album [20]. Each participant underwent a face-to-face interview conducted by a trained interviewer, and these sessions were audio-recorded with the participant’s consent, serving as a quality control measure.

### 2.4. Development and Administration of FFQ

The validated FFQ from the MANS 2014 [19], originally designed for general Malaysian adults aged between 18 and 59 years old, was adapted to reflect the dietary habits, portion sizes, and food items typical of the target population, ensuring alignment with the study objectives. The MyFCD [19] and a local food album [20] were referenced for this adaptation. The adapted FFQ consists of 13 food groups covering 165 food items known for their high salt content or frequent consumption. Adjustments included (1) reordering the FFQ to prioritize key food items, such as sauces or flavorings, which constitute significant dietary sources of sodium for Malaysian adults; (2) replacing standard portion sizes for some food items with commonly used household measures; (3) adding food pictures to facilitate participants’ self-administration of the FFQ; (4) incorporating Chinese translations to broaden accessibility; and (5) omitting alcoholic beverages due to their lack of relevance to the study outcome measures, minimal consumption within the study population, and exclusion of individuals with excessive alcohol use from the study. The adapted FFQ was reviewed by a panel of experts in nutrition and public health to assess its relevance and completeness in capturing the dietary patterns of the target population prior to its employment in this study.

During the baseline visit, participants were instructed to complete the FFQ. They were asked to recall the frequency of consumption (daily, weekly, or monthly) of each food item during the previous one-month period and estimate the portion size they typically consumed each time.

### 2.5. Statistical Analysis

Descriptive statistics were used to summarise participant characteristics and dietary sodium intake data. Median and interquartile range (IQR) were calculated instead of mean ± standard deviation (SD) due to a violation of the normality assumption. Gender differences were assessed using the Mann–Whitney U test for numerical variables and the Pearson Chi-Square or Fisher Exact test for categorical variables.

To evaluate the validity of alternative methods (spot urine, 1-day 24DR and FFQ) compared to the gold standard 24-h urine collection, the bias, Spearman correlation coefficient (SCC), intraclass correlation coefficient (ICC), and Bland–Altman (BA) analysis were used. Bias was calculated as the difference between the sodium measures derived from alternative methods and the 24-h urine collection. The SCC was used to determine the strength of the relationship between the methods. An SCC value < 0.3 indicated a weak correlation, 0.3 to 0.39 a moderate correlation, 0.4 to 0.69 a strong correlation, and ≥0.7 a very strong correlation. ICC values of less than 0.50, between 0.50 and 0.75, between 0.75 and 0.90, and greater than 0.90 were interpreted as poor, moderate, good, and excellent [21]. In the BA plots, the mean difference (bias) indicated whether one method tended to overestimate or underestimate sodium intake, while the limits of agreement (LoA), defined as Mean ± 1.96 SD, illustrated the extent of agreement between the two sodium intake assessment methods.

All statistical analyses were performed using R software version 4.1.2 (R Foundation for Statistical Computing, Vienna, Austria). The level of statistical significance was set at *p* < 0.05.

## 3. Results

### 3.1. Sociodemographic, Anthropometry, and Clinical Characteristics

The sociodemographic, anthropometry, and clinical characteristics of the participants included for analysis are presented in Table 1. The validation sample (*n* = 65) was 41.5% men, and the median age was 61 years old (IQR 11). Approximately 66% were Malays and 34% were Chinese. Most of the participants had completed secondary school education (53.8%) and had high levels of physical activity (44.6%) and normal cognition (95.4%).

All participants had diagnosed or self-reported elevated BP (prehypertension or hypertension) with median systolic BP 136 (IQR 24 mmHg) and diastolic BP 80 (IQR 13 mmHg). The proportion of hypertensive people in the validation cohort was 61.6% (*n* = 40), with 55.5% and 65.8% for men and women, respectively. Of those with hypertension, 42.5% (*n* = 17) were receiving prescribed antihypertensive medication.

The majority of the participants were overweight (83.1%). Higher BMI was observed more often in women (Median 27.4; IQR 6.1 kg/m^2^) than in men (Median 24.5; IQR 6.2 kg/m^2^) (*p*-value = 0.042). Waist circumference for men (Median 91.5; IQR 10.5 cm) and women (Median 89.6; IQR 13.6 cm) was higher than the recommendations of WHO/IASO/IOTF 2000, with 59.3% of men and 89.5% of women having abdominal obesity.

### 3.2. Biochemistry Data of 24-h Urine Collection and Salt Intake

The 24-h urinary biochemistry data and dietary salt intake are presented in Table 2. Overall, the median 24-h urine volume was 1386.2 (IQR 1257.8) mL, and the median collection time was 24 (IQR 1) hours. The median 24-h urinary sodium excretion measured via the reference method was 2615.5 (IQR 2182.4) mg/day. A longer duration of urine collection was observed for the 24-h urine samples of women (Median 24.4; IQR 1.6 h) than of men (Median 24.0; IQR 1.1 h) (*p*-value = 0.045). Men’s urine samples had significantly higher creatinine concentrations (Median 10.2; IQR 9.9 mmol/day) than those of women (Median 5.8; IQR 3.0 mmol/day) (*p*-value = 0.034).

The median salt intake was equivalent to 6.6 (IQR 5.5) g/day. There was no significant difference in salt consumption between men and women. Overall, less than one-third (*n* = 20; 30.8%) of participants had a salt intake that met the WHO’s recommendation of <5 g/day for salt intake [22].

### 3.3. Relative Validity of Alternative Methods for Estimating Dietary Sodium Intake

The relative validity of spot urine, 1-day 24DR, and FFQ compared to the 24-h urine collection for estimating dietary sodium consumption in middle-aged and older adults with elevated blood pressure is summarized in Table 3. Comparing the alternative methods to the reference standard, the mean biases observed in the 24-h urinary sodium excretion estimates were as follows: a bias of 33.3 mg/day (95% CI: −606.4 to 673.0 mg/day) for the INTERSALT equation using spot urine, a bias of 73.4 mg/day (95% CI: −696.9 to 843.7 mg/day) for the 1-day 24DR, and a bias of 441.6 mg/day (95% CI: −120.1 to 1003.3 mg/day) for the Tanaka equation applied to spot urine; the Kawasaki equation showed a considerably larger bias of 1414.0 mg/day (95% CI: 802.6 to 2025.3 mg/day). A negative mean bias was observed for FFQ (Mean difference −287.0 mg/day; 95% CI −1058.9 to 485.0 mg/day).

The SCC between estimated and measured 24-h urinary sodium excretion was 0.24 for spot urine collection with the Kawasaki equation, 0.23 for the Tanaka equation, 0.20 for the FFQ, 0.007 for 1-day 24DR, and −0.03 for the INTERSALT equation. Apart from the INTERSALT equation, which displayed a relatively small negative correlation, all other alternative methods exhibited positive correlations with 24-h urine collection.

The ICC values for the spot urine collection with the Kawasaki equation, Tanaka equation and FFQ were 0.12 (95% CI −0.08 to 0.328), 0.118 (95% CI −0.13, 0.34), and 0.07 (95% CI −0.17 to 0.31) (all *p*-values > 0.05), respectively. The ICC value for the INTERSALT equation was −0.11 (95% CI −0.35 to 0.14), and the ICC value for the 24DR was −0.06 (95% CI −0.31 to 0.19).

**Figure 2:**
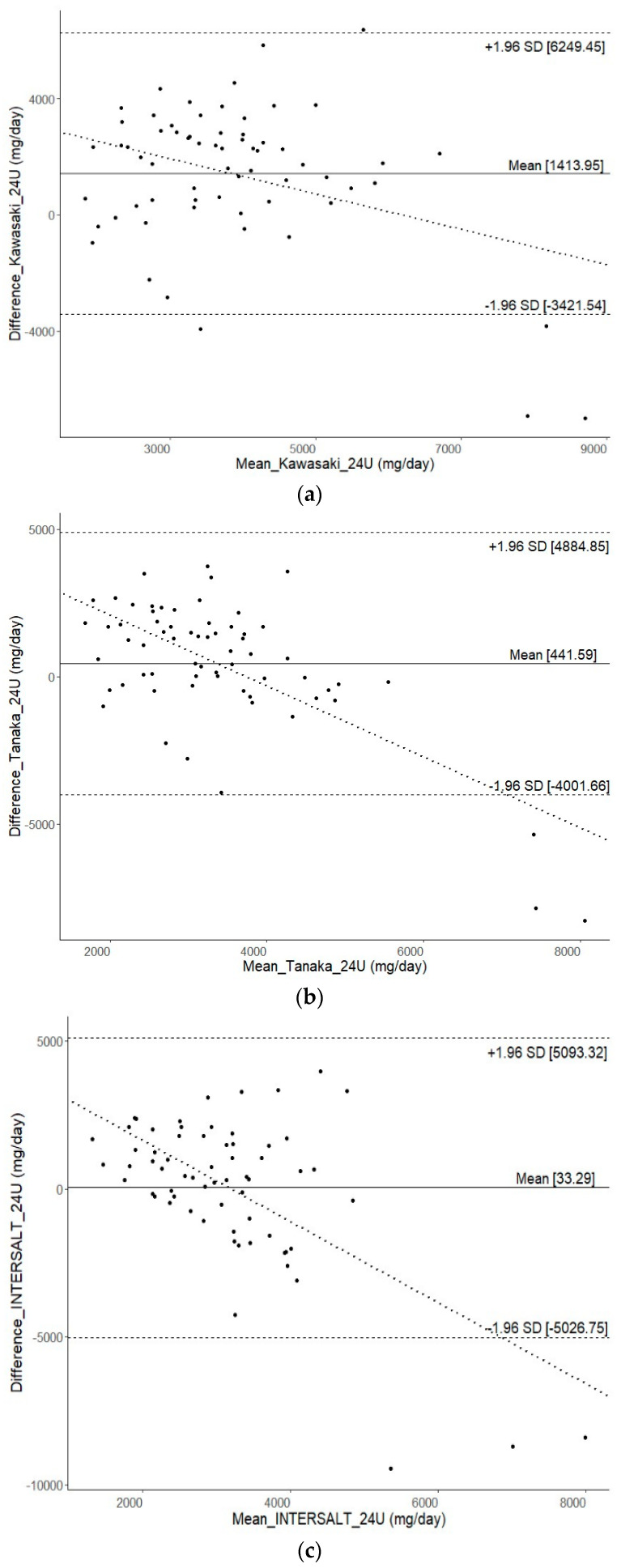

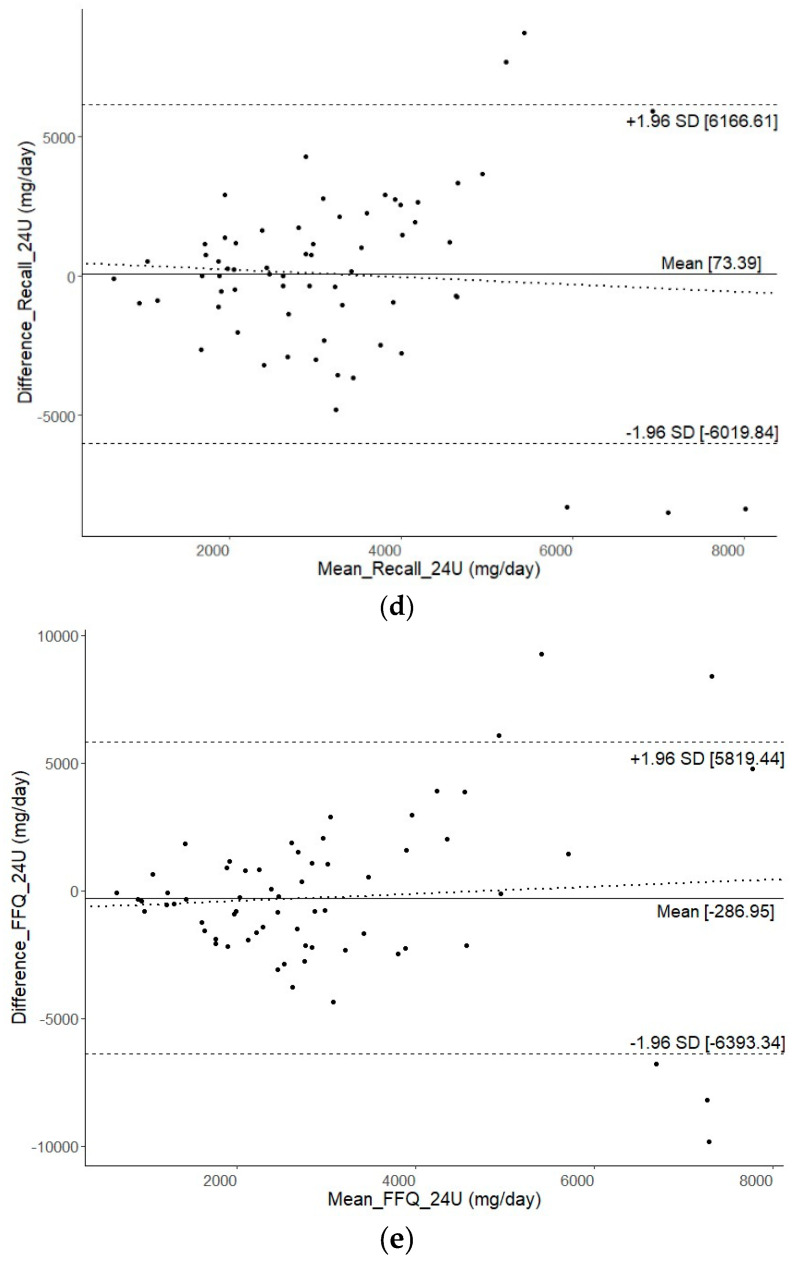
Bland–Altman plots of measured 24-h urinary sodium excretion (mg/day) versus the spot urine with associated equations [(**a**) Kawasaki; (**b**) Tanaka; (**c**) INTERSALT, (**d**) 1-day 24DR, and (**e**) FFQ]. The horizontal axis represents the mean [(estimated values + measured value)/2], and the vertical axis represents difference (estimated values—measured values). The solid line represents the mean of difference, the dashed lines represent the 95% limit of agreement of mean, and the dotted line represents the linear regression line.

BA plots (Figure 2) illustrate a reasonable level of agreement between alternative methods and 24-h urine collection, although there were very few individuals who fell outside the LoA. However, a wide range of LoA was observed across all alternative methods. The widest range of LoA were found for 1-day 24DR (LoA −6019.8 to 6166.6 mg/day), followed by the FFQ (LoA −6393.3 to 5819.4 mg/day), spot urine with the INTERSALT equation (LoA −5026.7 to 5093.3 mg/day), the Kawasaki equation (LoA −3421.5 to 6249.5 mg/day), and the Tanaka equation (LoA −4001.7 to 4884.8 mg/day).

## 4. Discussion

This study assessed the validity of alternative sodium intake estimation methods against the 24-h urine collection in middle-aged and older adults with elevated BP. The findings reveal that the participants’ median daily salt intake of 6.6 g (equivalent to 2.6 g of sodium) exceeded the WHO’s recommended maximum of 5 g/day [22]. This corroborates earlier studies in Malaysia, indicating adults’ average sodium consumption of 1.9 g to 3.4 g/day [23,24,25,26]. Factors contributing to these high sodium intakes may include dietary habits, cultural preferences, food availability, and the presence of sodium-rich processed foods in the Malaysian diet. The Malaysian Community Salt Survey (MyCoSS) study of salt intake shows that 79% of Malaysians are consuming 7.9 g (1.6 teaspoons) of salt, or 3167 mg of sodium, per day [25]. However, this amount is above the intake recommended by the WHO, which is less than 5 g of salt per day, or less than 2 g of sodium per day, for an average adult. According to the Recommended Nutrient Intake (RNI) 2017, the sodium requirement is 1.5 g per day for an average Malaysian adult aged 19 years old and above [27]. Elevated sodium consumption raises concerns about cardiovascular risks, necessitating robust public health initiatives to reduce intake and prevent associated health issues.

Validity assessment considered bias, SCC, ICC, and LoA between measured and estimated 24-h urinary sodium excretion. Despite reasonable agreement, substantial bias, weak SCC and ICC, and wide LoA across all alternative methods collectively highlight challenges in accurately estimating sodium intake in middle-aged and older adults with elevated blood pressure.

Spot urine equations derived from younger (<60 years) [11,28], healthy [11,12,28] adults may not generalize to this population, particularly those on blood pressure medication. These equations may not account for hypertension’s impact on sodium handling, contributing to estimation limitations. Negative SCC and ICC values for the INTERSALT equation are likely due to its Western origin, inadequately addressing Malaysia’s multiethnic context and its diverse dietary, cultural, genetic, and physiological factors.

Generally, the spot urine collection method showed better SCC and ICC compared to dietary surveys, while dietary methods had a smaller bias but wider LoA. This suggests stronger associations and agreements between estimated and measured 24-h urinary sodium excretion values using spot urine. Short-term variations in sodium intake may contribute to better SCC and ICC for spot urine, reflecting its direct sodium excretion capture. Dietary methods, prone to recall bias and portion estimation variability, exhibited weaker SCC and ICC but a smaller bias. While dietary surveys may better capture overall sodium intake trends, wider LoA indicates that individual estimations may significantly deviate from measured values due to varying dietary choices and habits.

The BA plots generated using the spot urine method with all tested equations consistently revealed underestimation, particularly for extremely high sodium intake, aligning with the previous literature [9,10,11,12]. This underlines limitations in accurately estimating 24-h urinary sodium excretion, especially at high intake levels. A “dilution effect” in the spot urine method may result from excessive sodium intake, increasing urine output and lowering concentration levels, impacting estimates. Concurrently, BA plots for dietary surveys indicated a magnification of bias with higher mean 24-h urinary sodium excretion levels. Interestingly, the bias direction for the 24DR and the FFQ diverged. The 24DR exhibited an increasing negative bias with higher sodium intake, suggesting possible under-reporting. This could be attributed to participants under-reporting sodium intake as their mean intake rises, possibly due to health-related concerns or social desirability bias. In such cases, participants may provide responses aligning with healthier dietary habits, particularly in the setting of a nutritional intervention trial. Conversely, over-reporting of sodium intake in the FFQ may occur due to recall bias or inaccuracies in estimating portion sizes. In such cases, individuals might overestimate their consumption of high-sodium foods, believing they are consuming larger portions than they are.

This validation study assessing sodium intake in middle-aged and older adults with elevated BP addresses an important research gap by focusing on this specific demographic group. While 24-h urine collection is considered the gold standard for assessing sodium intake, it is resource-intensive and burdensome for participants, making it less practical in large-scale studies, especially among older adults. This study evaluated three alternative methods: spot urine collection with associated equations (Kawasaki, Tanaka, and INTERSALT), 24DR, and the FFQ. By comparing these methods to the gold standard, a comprehensive assessment of their relative validity could be offered, allowing researchers and healthcare professionals to make informed choices about the most suitable method for assessing sodium intake in middle-aged and older adults with elevated BP. This information is essential for the development of effective dietary interventions and public health strategies aimed at reducing sodium intake and improving cardiovascular health in this specific demographic group.

Nonetheless, it is important to acknowledge the limitations of this study. This study employed a 1-day 24DR instead of a 3-day 24DR for the enhanced response rate and facilitated administration, particularly given the older adult demographic of the study participants. Previous research has reported that a 3-day 24DR was perceived as burdensome by participants due to the requirement for fixed interview times and the repetitive nature of the questions, potentially impacting response rates and the quality of data collected [29]. However, it is noteworthy that while a 1-day 24DR can offer insights into daily intake, it may not capture the day-to-day variations in dietary patterns that a 3-day 24DR, incorporating both weekdays and a weekend day, would provide. Moreover, this study’s findings may have limited generalizability to the wider population due to the relatively small sample size and the specific inclusion criteria targeting individuals with elevated BP. Consequently, these results might not be directly transferable to individuals with normotensive BP readings or those with different health profiles.

## 5. Conclusions

Our study highlights the challenges involved in accurately estimating dietary sodium intake among middle-aged and older adults with elevated blood pressure. Spot urine collection, while showing stronger associations with the reference method of 24-h urine collection, also tends to introduce more significant bias. In contrast, dietary surveys exhibit less bias but come with wider limits of agreement. To enhance the accuracy of dietary sodium intake estimation within this demographic group, it is recommended to consider a combination of methods or further refinement of existing methodologies. A holistic approach that integrates both the spot urine collection method and dietary surveys could offer a more comprehensive perspective, offering both strong associations and reduced bias. Public health efforts aimed at reducing sodium intake remain crucial, given the high prevalence of hypertension and excessive sodium consumption in adults. Further research with larger samples is needed to develop more reliable approaches for assessing sodium intake in this population.

## Figures and Tables

**Table 1:** The sociodemographic, anthropometry, and clinical characteristics of the participants included in this study.

Characteristics	Total (*n* = 65)	Men (*n* = 27)	Women (*n* = 38)	*p*-Value *
Age, years	61.0 (11.0)	61.0 (10.0)	61.5 (13.0)	0.627
Ethnicity, *n* (%)				0.647
Malay	43 (66.2)	17 (63.0)	26 (68.4)	
Chinese	22 (33.8)	10 (37.0)	12 (31.6)	
Educational attainment, *n* (%)				0.468
No formal education	6 (9.2)	1 (3.7)	5 (13.2)	
Primary	22 (33.8)	8 (29.6)	14 (36.8)	
Secondary	35 (53.8)	17 (63.0)	18 (47.4)	
Tertiary	2 (3.1)	1 (3.7)	1 (2.6)	
Hypertension ^a^ status, *n* (%)				0.474
Prehypertensive	25 (38.5)	12 (44.4)	13 (34.2)	
Hypertensive with medication	17 (26.2)	5 (18.5)	12 (31.6)	
Hypertensive without medication	23 (35.4)	10 (37.0)	13 (34.2)	
Blood pressure, mmHg				
Median systolic	136.0 (24.0)	139.0 (33.0)	136.0 (20.0)	0.754
Median diastolic	80.0 (13.0)	79.0 (15.0)	80.5 (11.0)	0.917
BMI ^b^, kg/m^2^	26.5 (6.7)	24.5 (6.2)	27.4 (6.1)	0.042
Normal, *n* (%)	11 (16.9)	8 (29.6)	3 (7.9)	0.041
Overweight, *n* (%)	54 (83.1)	19 (70.4)	35 (92.1)	
Waist circumference, cm	90.5 (12.9)	91.5 (10.5)	89.6 (13.6)	0.543
Central obesity ^c^, *n* (%)	50 (76.9)	16 (59.3)	34 (89.5)	0.004
Diabetes ^d^, *n* (%)	7 (10.8)	2 (7.4)	5 (13.2)	0.690
Current smoker, *n* (%)	10 (15.4)	9 (33.3)	1 (2.6)	0.001
Alcohol user, *n* (%)	5 (7.7)	3 (11.1)	2 (5.3)	0.642
MMSE ^e^ score	26 (4)	26 (4)	26 (4)	0.564
Normal, *n* (%)	62 (95.4)	25 (92.6)	37 (97.4)	0.565
Cognitive impaired, *n* (%)	3 (4.6)	2 (7.4)	1 (2.6)	
Physical activity ^f^				
MET-mins/week	2320 (4560)	3600 (5880)	1820 (3120)	0.543
Low	17 (26.2)	6 (22.2)	11 (28.9)	0.319
Moderate	19 (29.2)	6 (22.2)	13 (34.2)	
High	29 (44.6)	15 (55.6)	14 (36.8)	

Data are presented as median (IQR, interquartile range) unless otherwise specified. ^a^ Prehyertension, median blood pressure ≥ 120/80 mmHg; hypertension, median blood pressure ≥ 140/90 mmHg at physical examination, or they were receiving antihypertensive medications or had a previous diagnosis for at least 2 weeks. ^b^ BMI, body mass index: normal, 18.5–22.9 kg/m^2^; overweight, ≥23 kg/m^2^; obese, ≥27.5 kg/m^2^. Classification is based on the Asian BMI guideline proposed by the WHO Western Pacific Region in 2000. ^c^ Waist circumference ≥ 90 cm in men and ≥80 cm in women, based on the modified National Cholesterol Education Program Adult Treatment Panel (NCEP ATP-III) cut-offs in Asians. ^d^ Self-reported. ^e^ The Malay version of Mini Mental State Examination serial 7 (M-MMSE-7) was used. For males, these scores were used: ≤23, cognitive impaired and ≥24, normal; for females, these scores were used: ≤19 cognitive impaired and ≥20 normal. ^f^ Assessed by the Global Physical Activity Questionnaire (GPAQ), which was used to calculate metabolic equivalents (MET)mins/week. Scores: <600, low; 600 ≤ score < 3000, moderate; ≥3000, high. * Differences between gender tested with the Mann–Whitney U (numerical variable), Pearson chi-square, or Fisher Exact (categorical variable) tests. Significant differences if *p* < 0.05.

**Table 2:** The 24-h urinary biochemistry and estimated dietary salt intake data.

Variable	Total (*n* = 65)	Men (*n* = 27)	Women (*n* = 38)	*p*-Value *
Urinary biochemistry				
Volume, mL	1386.2 (1257.8)	1589.7 (1493.1)	1269.6 (1079.2)	0.543
Duration of collection, h	24.0 (1.0)	24.0 (1.1)	24.4 (1.6)	0.045
Sodium excretion, mg/day	2615.5 (2182.4)	2477.4 (1910.7)	2659.3 (2410.4)	0.917
Creatinine concentration, mmol/day	6.3 (5.6)	10.2 (9.9)	5.8 (3.0)	0.034
Dietary salt intake				
Salt intake ^a^, g/day	6.6 (5.5)	6.3 (4.9)	6.8 (6.1)	0.917
Met WHO’s recommendation of <5 g/day salt intake, *n* (%)	20 (30.8)	7 (25.9)	13 (34.2)	0.476

Data are presented as median (IQR, interquartile range) unless otherwise specified. ^a^ Measured via 24-h urine collection. Conversion from sodium to salt was made by multiplying a factor of 2.542. * Differences between gender tested with the Mann–Whitney U (numerical variable), Pearson chi-square, or Fisher Exact (categorical variable) tests. Significant differences existed if *p* < 0.05.

**Table 3:** Relative validity of spot urine, 1-day 24DR, and FFQ versus 24-h urine collection for estimating dietary sodium consumption in middle-aged and older adults with elevated blood pressure.

Method	* Mean Difference (mg/Day) (95% CI)	SCC	ICC (95% CI)
24-h urine (reference)	Reference	Reference	Reference
Spot urine (Kawasaki equation)	** 1414.0 (802.6 to 2025.3)	0.24	0.12 (−0.08 to 0.32)
Spot urine (Tanaka equation)	441.6 (−120.1 to 1003.3)	0.23	0.11 (−0.13 to 0.34)
Spot urine (INTERSALT equation)	33.3 (−606.4 to 673.0)	−0.03	−0.11 (−0.35 to 0.14)
1-day 24-h dietary recall	73.4 (−696.9 to 843.7)	0.01	−0.06 (−0.31 to 0.19)
Food Frequency Questionnaire	−287.0 (−1058.9 to 485.0)	0.20	0.07 (−0.17 to 0.31)

CI, confidence interval; ICC, intraclass correlation coefficient; SCC, Spearman correlation coefficient. * Differences between the alternative method and 24-h urine collection method were calculated with a paired *t*-test. ** *p*-value < 0.001.

## Data Availability

The data presented in this study are available on request from the corresponding author. The data are not publicly available due to privacy concerns.
